# Exosomes Generated by Normal Peritoneal Cells Driven to Senescence by Carboplatin and Paclitaxel Awaken Dormant Ovarian Cancer Cells and Support Their Growth Reinitiation In Vitro

**DOI:** 10.3390/cancers18091346

**Published:** 2026-04-23

**Authors:** Szymon Rutecki, Adrianna Krawiec, Agnieszka Leśniewska-Bocianowska, Julia Matuszewska, Eryk Naumowicz, Sebastian Szubert, Krzysztof Książek, Justyna Mikuła-Pietrasik

**Affiliations:** 1 Department of Pathophysiology of Ageing and Civilization Diseases, Poznań University of Medical Sciences, Święcickiego 4 Str., 60-781 Poznań, Poland; akrawiec@ump.edu.pl (A.K.); abocianowska@ump.edu.pl (A.L.-B.); matuszewskaj@ump.edu.pl (J.M.); jmikula@ump.edu.pl (J.M.-P.); 2 Doctoral School, Poznan University of Medical Sciences, Bukowska 70 Str., 60-812 Poznań, Poland; 3 General Surgery Ward, Medical Centre HCP, 28 Czerwca 1956 r. 223/229 Str., 61-485 Poznan, Poland; eryknaumowicz777@gmail.com; 4 Department of Gynecology, Division of Gynecologic Oncology, Poznan University of Medical Sciences, Polna 33 Str., 60-535 Poznań, Poland; sszubert@ump.edu.pl; 5 Faculty of Medical Sciences, Prince Mieszko I Poznan Medical University of Applied Sciences, Bułgarska 55 Str., 60-320 Poznań, Poland; k.ksiazek@pam.poznan.pl

**Keywords:** cellular senescence, epithelial ovarian cancer, dormant cells, tumor relapse

## Abstract

**Background**: Recurrence poses a major challenge in epithelial ovarian cancer (EOC), often occurring despite optimal first-line therapy. Dormant cancer cells are believed to play a key role, yet the mechanisms driving their reactivation remain unclear. This study examined whether exosomes released by normal peritoneal mesothelial cells (PMCs) and fibroblasts (PFBs) undergoing iatrogenic senescence after carboplatin and paclitaxel exposure contribute to EOC recurrence. **Methods and Results**: Senescent PMCs and PFBs secreted markedly more exosomes, identified by CD9, CD63, and CD81, compared with young cells. Exosomes from both cell types more effectively reactivated dormant EOC cells (pEOCs, A2780, OVCAR-3, SKOV-3) than non-exosomal medium constituents. Importantly, senescent PMC-derived exosomes most strongly reactivated pEOCs and SKOV-3, whereas those from senescent PFBs exerted greater effects on pEOCs, OVCAR-3, and SKOV-3. Kinetic studies of exosome internalization revealed that this process was generally more efficient in the presence of exosomes derived from senescent cells compared with those from young donor cells. Compositional analysis revealed distinct profiles between young and senescent exosomes compared in two variants: young PMCs/senescent PMCs and young PFBs/senescent PFBS. Senescent PMC exosomes displayed reduced miR-210-3p, miR-409-3p, and miR-421, alongside elevated MMP1, MMP3, and VEGF, while senescent PFB exosomes showed increased amphiregulin and osteopontin but lower MMP1, MMP3, TIMP1, bFGF, VEGF, and HGF. Functionally, senescent PMC exosomes enhanced pEOC migration, invasion, and spheroid formation, and induced the expression of CCL11 and ABCB1. Senescent PFB exosomes promoted migration and upregulated CCL11, TGF-β1, BIRC5, and CHEK1. **Conclusions**: These findings suggest that therapy-induced senescence in peritoneal cells may contribute to EOC recurrence by reactivating dormant tumor cells through exosomal signaling.

## Simple Summary

This study investigates the mechanism that may contribute to ovarian cancer recurrence, which represents a major clinical challenge that remains poorly understood. This work demonstrates that normal peritoneal cells driven into senescence by carboplatin and paclitaxel release exosomes capable of reactivating dormant ovarian cancer cells and proliferation in vitro. These findings suggest that chemotherapy-induced senescence in the tumor microenvironment may inadvertently create conditions favorable for tumor regrowth, thereby offering new insight into relapse biology.

## 1. Introduction

Epithelial ovarian cancer (EOC) is among the most lethal malignancies, largely due to its high propensity for recurrence. Epidemiological studies estimate that up to 70% of patients with EOC experience disease relapse following initial therapy [1]. This reactivation significantly worsens prognosis, primarily because recurrent tumors often acquire resistance to conventional chemotherapeutic agents, limiting the effectiveness of subsequent treatment strategies [2]. Emerging evidence suggests that cancer relapse may be driven by the reactivation of so-called dormant cancer cells. These cells represent a subpopulation of disseminated or residual tumor cells that survive initial treatment but enter a temporary state of growth arrest in response to unfavorable microenvironmental conditions. Such conditions may include a scarcity of mitogenic signals, insufficient nutrient or oxygen supply, or the suppressive actions of immune surveillance mechanisms [3]. The duration of cellular dormancy can vary significantly, ranging from several months to several years, depending on the tumor type, microenvironmental cues, and patient-specific factors [4]. Crucially, dormant cancer cells retain the capacity to resume proliferation and contribute to tumor regrowth when exposed to permissive tissue conditions [5]. Despite growing recognition of their clinical significance, the precise molecular and microenvironmental signals that trigger the exit from dormancy remain poorly defined. Understanding these mechanisms is crucial for developing therapeutic strategies that aim to prevent relapse and overcome chemoresistance in EOC.

In our previous study, we found that normal peritoneal mesothelial cells (PMCs) and fibroblasts (PFBs) exposed to a combination of carboplatin and paclitaxel undergo therapy-induced senescence, developing a pro-tumorigenic phenotype. Importantly, the conditioned medium produced by these senescent cells stimulates the proliferation, migration, and invasion of both established and primary EOC cells [6]. Furthermore, we developed a reliable method to investigate the induction and termination of EOC cell dormancy by sequentially adding and removing serum from the culture conditions [7]. In the current study, we built on these experimental protocols to explore whether therapy-induced senescent PMCs and PFBs may play a role in EOC relapse by generating exosomes that could activate dormant cancer cells and trigger their further growth.

## 2. Materials and Methods

### 2.1. Cells Cultures

Peritoneal mesothelial cells (PMCs) and peritoneal fibroblasts (PFBs) were isolated from the omentum of non-oncologic patients undergoing abdominal surgery, and they were maintained according to the protocol outlined in reference [8]. The ovarian cancer cell lines A2780 and SKOV-3 were sourced from the ECCC in Porton Down, Wiltshire, UK, while OVCAR-3 cells were obtained from ATCC in Rockville, MD, USA. Details regarding the culture conditions for these cell lines can be found in reference [9]. Additionally, primary epithelial ovarian cancer cells (pEOC) were isolated from chemotherapy-naïve patients with high-grade serous tumors; their isolation, identification, and culturing procedures are described in the reference [10]. Human ovarian surface epithelial cells (HOSEpiC) were maintained under standard cell culture conditions in Poly-L-Lysine-coated T-flasks, using Ovarian Epithelial Cell Medium (OEpiCM). Both the HOSE cells and the necessary poly-L-lysine and culture medium were obtained from ScienCell Research Laboratories in Carlsbad, CA 92008, USA. The study received approval from an institutional ethics committee (consent numbers 578/18 and 539/21), and all participating patients provided informed consent.

### 2.2. Drug-Dependent Senescence Induction

Senescence in PMCs and PFBs was induced by exposing the cells to two different compound combinations: 50 µM carboplatin (CPT) and 25 nM paclitaxel (PCT) for PMCs, and 25 µM CPT and 10 nM PCT for PFBs, as previously described [6]. The exposure took place in standard growth medium at 37 °C for 72 h. Following this period, the cells were washed with Hank’s Balanced Salt Solution (HBSS) and then maintained in growth medium for an additional 24 h to allow for recovery. After recovery, the cells were re-passaged and incubated for another 7 to 10 days [6].

### 2.3. Cell Dormancy Induction and Termination

Cancer cell dormancy was induced by serum starvation, as described in [7] with minor modifications. Cells were cultured in 6-well plates (surface area 9.6 cm^2^ per well) at a density of 2 × 10^5^ cells per well at approximately 70% confluency and then washed with PBS before being placed in serum-free medium (SFM) in a volume of 2 mL per well for 24 to 72 h. To terminate dormancy, serum-free growth medium supplemented with exosomes from 500,000 young or senescent cells, in a volume of 2 mL per well, was reintroduced for 72 h. Alternatively, exosomes derived from varying numbers of young and senescent cells were used, maintaining a consistent total number of exosomes for 72 h. In some experiments, the effects of conditioned medium from PMCs and PFBs were compared with those of exosomes isolated from these media when added to fresh, serum-free medium. The expression of the Ki67 proliferative antigen served as an indicator of the reinitiation of growth in dormant cells.

### 2.4. Quantification of Ki67 Expression

Cells were fixed using 4% formaldehyde and then permeabilized with 0.3% Triton X-100 (Merck, Darmstadt, Germany) in PBS for 10 min at room temperature (RT). To block non-specific binding, the cells were treated with a solution containing 1% BSA (Merck, cat no. A3059), 5% goat serum (Merck), and 0.3% Triton-X 100 in PBS for 1 h at RT. Following this, the cells were incubated overnight at 4 °C with a rabbit anti-Ki67 monoclonal antibody (D3B5, Cell Signaling Technology, Boston, MA, USA, cat. no. 9129T) at a dilution of 1:400 in blocking buffer. Afterward, they were treated with Goat anti-Rabbit IgG Alexa Fluor 488 (Invitrogen, Waltham, MA, USA, cat no. A11008) at a dilution of 1:500 in PBS for 1 h at RT. The samples were then mounted using DAPI fluoroshield (Abcam, Cambridge, UK, cat no. ab104139) and imaged with an Axio Vert.A1 microscope (Carl Zeiss, Jena, Germany). A total of 200 cells per sample were evaluated.

### 2.5. Exosome Isolation, Identification, and Quantification

Exosomes were isolated from the conditioned medium using the Total Exosome Isolation Reagent (Invitrogen) according to the manufacturer’s instructions.

Identification of exosomes labeled at room temperature for 1 h with anti-human CD9 PE (Sony Biotechnology, San Jose, CA, USA, cat. no. 2160530), CD63 Monoclonal Antibody (MEM-259) PE (Invitrogen, cat. no. MA1-19650), and CD81 Monoclonal Antibody (M38) PE (Invitrogen, cat. no. MA1-10292) was carried out using an Amnis CellStream flow cytometer (Lumiex Corporation, Austin, TX, USA). Antibodies were suspended in Dulbecco’s Phosphate-Buffered Saline (PBS, Biowest, Nuaillé, France, cat. no. MS02PE1001) at a dilution of 1:100, and centrifuged at 15,000× *g* at 4 °C for 20 min. Incubation with exosomes collected from 100,000 cells was performed in a 96-well plate for 1 h at room temperature in the dark. Control groups included exosomes not incubated with antibodies, exosomes labeled with antibodies with the addition of 0.01% Triton-X (where extracellular vesicle degradation occurred), and the PE Mouse IgG1 Isotype Control (Sony Biotechnology, cat. no. 2600570). Quantitative analysis of the results was performed using the CellStream Analysis software 1.3.384 (Lumiex Corporation, Austin, TX, USA). Results are expressed as the number of marker-positive events per 60 s acquisition, allowing direct comparison between young and senescent cell populations.

Scanning electron microscopy images of the exosome samples coated with a 5 nm layer of iridium were captured using a Zeiss Sigma microscope (Carl Zeiss) equipped with an objective aperture of 30 µm. Samples containing exosomes in 2% glutaraldehyde were pulse-vortexed and then applied onto cover slips in a volume of 10 μL. The cover slips were dried at room temperature for 30 min. The preparation was fixed using a series of alcohols with successive concentrations of 50%, 70%, 90%, and 99%, for 5 min each. After the final incubation, the excess alcohol was removed, and the preparations were left to dry completely overnight at room temperature.

For functional experiments, two normalization strategies were employed: (1) ‘Cargo’ experiments: equal numbers of exosomes (based on flow cytometry counts) were applied to recipient cells, enabling assessment of cargo-specific effects independent of production differences; (2) ‘Quantity’ experiments: exosomes derived from equal numbers of donor cells (500,000 young or senescent cells) were applied, reflecting the natural production differences between young and senescent cells. This dual approach allowed us to distinguish between effects attributable to exosome quantity versus molecular composition.

### 2.6. Exosome Internalization Analysis

Exosomes derived from both young and senescent PMCs and PFBs were labeled with a conjugated CD81 antibody (PE) (Invitrogen, cat. no. MA1-10292) at a dilution of 1:200. These exosomes were incubated with the CD81 antibody for 1 h at room temperature in the dark. Internalization was performed on a 96-well plate. The labeled exosomes from 200,000 cells per well were then administered to primary EOC cells (pEOCs) as well as established EOC cell lines. Incubation occurred at four different time points: 10 min, 30 min, 1 h, and 2 h. After incubation, excess unbound exosomes were washed away with PBS, and the samples were fixed with 4% paraformaldehyde (PFA). Cells were stained with Cell Tracker Green BODIPY (Invitrogen, cat. no. C2102) according to the manufacturer’s protocol. The level of exosomes internalization was assessed by measuring fluorescence intensity using a fluorescence microscope, the APEXVIEW APX100 (Evident Europe GmbH, Hamburg, Germany).

### 2.7. Analysis of Exosomal Proteins, mRNA, and miRNA

To quantify exosomal proteins, we homogenized the exosomes using a Bandelin Sonoplus sonicator (Bandelin, Germany). We then conducted a proteomic analysis using a Human custom array (H20 STI, RayBiotech Life, Norcross, GA, USA; cat. no. QAH-CUST) according to the manufacturer’s instructions.

Total RNA isolation was performed using the Total Exosome RNA & Protein Isolation Kit (Invitrogen) according to the manufacturer’s instructions. Gene expression analysis was performed using the RT^2^ Profiler PCR Array (QIAGEN Sciences, Germantown, MD, USA) for the following genes: GAPDH, IL6, CXCL8, CXCL12, CXCL1, CCL2, TGFB1, TIMP1, SPP1, MMP1, MMP2, and LPA. The analysis was performed using the recommended reagents from QIAGEN Sciences, specifically the RT^2^ First Strand Kit and the RT^2^ SYBR Green qPCR Mastermix. In other experiments, RNA isolation was performed using the InviTrap Spin Universal RNA Mini Kit (Invitek Molecular GmbH, Berlin, Germany; cat. no. 1060100200) according to the manufacturer’s instructions. Reverse transcription was carried out with the Transcriptor First Strand cDNA Synthesis Kit (Roche Diagnostics GmbH, Mannheim, Germany; cat. no. 4897030001) in a Thermal Cycler T100 (Bio-Rad Laboratories Inc., Hercules, CA, USA), adhering to the manufacturer’s protocol. Gene expression analyses for GAPDH, BIRC5, ABCB1, ABCC4, CHEK1, CHEK2, CCL11, TIMP1, HGF, VEGF, and TGFB1 were conducted according to the manufacturer’s guidelines for the FastStart Essential DNA Green Master kit (Roche Diagnostics GmbH; cat. no. 6402712001) and were analyzed using the LightCycler^®^ 96 Instrument as previously described.

miRNA analysis was performed using the TaqMan™ Advanced miRNA cDNA Synthesis Kit (Applied Biosystems™, Waltham, MA, USA) and the TaqMan™ Fast Advanced Master Mix (Applied Biosystems™) according to the manufacturer’s instructions. The TaqMan™ probes used in the experiment were obtained from the Thermo Fisher Scientific (Waltham, MA, USA) library and included: hsa-miR-484, hsa-miR-27b-5p, hsa-miR-27b-3p, hsa-miR-105-5p, hsa-miR-409-5p, hsa-miR-409-3p, hsa-miR-612, hsa-miR-4748, hsa-miR-614, hsa-let-7a-5p, hsa-miR-1299, hsa-miR-421, hsa-miR-210-3p, hsa-miR-543, hsa-miR-323a-3p, and hsa-miR-3689d. The analysis of mRNA and miRNA was conducted using the LightCycler^®^ 96 Instrument from Roche Diagnostics GmbH, Germany. The selection of microRNAs was conducted using the mirDIP: MicroRNA Data Integration Portal (https://ophid.utoronto.ca/mirDIP/; accessed on 30 March 2026). Functional associations were analyzed using the miRBase MicroRNA Database (https://www.mirbase.org/; accessed on 30 March 2026) and the miRDB (https://mirdb.org/; accessed on 30 March 2026) databases.

### 2.8. Cancer Cell Progression Analysis

Cell proliferation was assessed using a 24 h protocol with the Cell Proliferation Kit I (PromoKine, Heidelberg, Germany). The migration assay was performed using ChemoTx migration chambers (Neuro Probe, Gaithersburg, MD, USA). The invasiveness of cancer cells treated with exosomes was evaluated using the Cell Invasion Assay (Basement Membrane), 96-well, 8 µm (Abcam, Cambridge, UK, cat. no. ab235697) according to the manufacturer’s instructions. The chemotactic factor in the ChemoTx migration chambers test and the Cell Invasion Assay was the basic culture medium supplemented with 2% exosome-depleted serum. For spheroid formation, 96-well plates (Thermo Fisher Scientific) were used and coated with 100 µL of Corning^®^ Matrigel^®^ Matrix for Organoid Culture (Corning Inc., New York, NY, USA). A total of 10,000 cells were seeded onto the Matrigel layer to allow the formation of a three-dimensional structure. Qualitative analysis of the spheroids was performed using an Olympus APEXVIEW APX100 microscope (Evident Europe GmbH, Hamburg, Germany). The size analysis of the 3D cell cultures was carried out using the Olympus cellSens APEX 4.2 software (Evident Europe GmbH).

### 2.9. Statistics

Statistical analysis was performed using GraphPad Prism 6.00 software (GraphPad Software, San Diego, CA, USA). The means were compared using the Wilcoxon matched-pairs test. The results are expressed as the mean ± SEM. Differences with a *p*-value < 0.05 were considered to be statistically significant.

## 3. Results

### 3.1. Senescent PMCs and PFBs Produce Higher Amounts of Exosomes Compared to Young Cells

**Figure 1:**
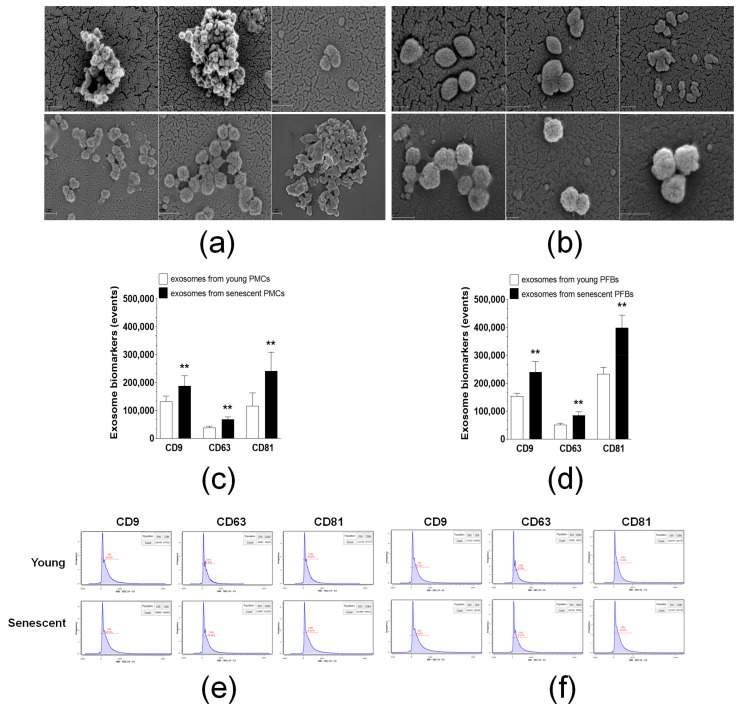
Characterization of exosomes derived from PMCs and PFBs. (**a**) Representative scanning electron microscopy (SEM) images of exosomes isolated from PMC-conditioned medium, showing rounded nanovesicles with diameters ranging from 40 to 180 nm. Scale bar = 200 nm. (**b**) Representative SEM images of exosomes isolated from PFB-conditioned medium, displaying similar morphological characteristics. Scale bar = 200 nm. (**c**) Quantitative flow cytometry ** *p* < 0.01 analysis of exosomes from young and senescent PMCs based on the expression of canonical exosomal markers CD9, CD63, and CD81. (**d**) Quantitative flow cytometry ** *p* < 0.01 analysis of exosomes from young and senescent PFBs. (**e**) Representative flow cytometry histograms illustrating the quantitative differences in CD9-, CD63-, and CD81-positive exosomes between young (gray) and senescent (red) PMCs. (**f**) Representative histograms for PFBs. ** *p* < 0.01 for comparisons against young cells. *n* = 8 donors per group; results expressed as mean ± SEM.

Exosomes were isolated by precipitation from conditioned medium produced by early-passage PMCs and PFBs, as well as from their counterparts induced into premature senescence by exposure toCPT and PCT, as previously described [6]. Scanning electron microscopy (SEM) analysis revealed that this method successfully yielded a homogeneous fraction of rounded nanomolecules, with sizes ranging from 40 to 180 nm (Figure 1a,b). Quantitative analysis using highly sensitive flow cytometry revealed that senescent peritoneal cells produce significantly more exosomes compared to young cells of equal quantity. Specifically, senescent PMCs released 39 ± 14%, 93 ± 17%, and 144 ± 57% more CD9-positive, CD63-positive, and CD81-positive exosomes, respectively, than their young counterparts (Figure 1c,e). For PFBs, senescent cells released 57 ± 23%, 75 ± 33%, and 83 ± 30% more CD9-positive, CD63-positive, and CD81-positive exosomes than young PFBs (Figure 1d,f). By averaging the values for all markers, we conclude that senescent PMCs generate 92% more exosomes, while senescent PFBs produce 72% more exosomes than their young counterparts.

### 3.2. Exosomes Awaken Dormant Cancer Cells More Efficiently than Non-Exosomal Medium Constituents

**Figure 2:**
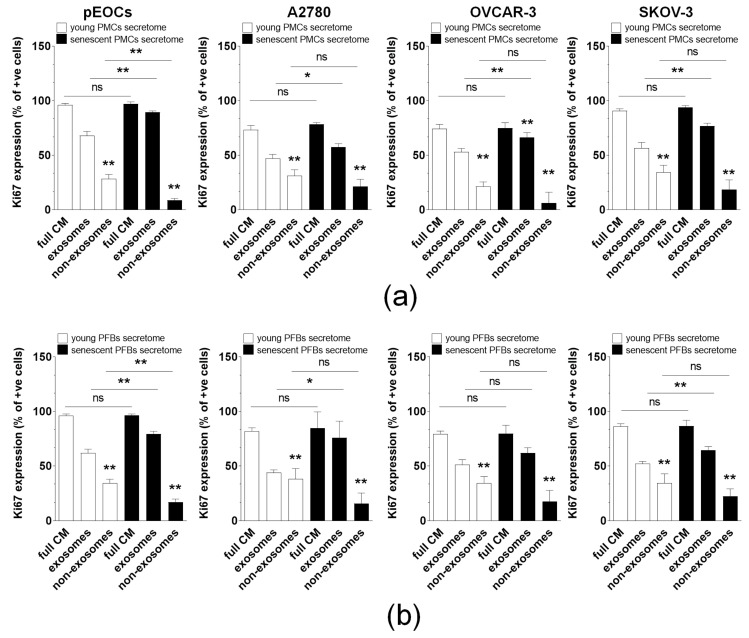
A comparative analysis of the potency of conditioned media, exosomes, and non-exosomal fraction in reactivating dormant ovarian cancer cells. The reawakening potential of secretomes from PMC (**a**) and PFB (**b**) was compared, which included both full conditioned medium and serum-free medium enriched with exosomes isolated from the conditioned medium. The impact of non-exosomal fraction was determined by subtracting the awakening potential induced by exosomes from that of the conditioned media. ** *p* < 0.01 when comparing with exosomes. Exosomes derived from PMCs and PFBs obtained from eight different donors were utilized in the experiments, with results presented as mean ± SEM. Statistical significance was defined as * *p* < 0.05, ** *p* < 0.01, while ns indicates non-significant differences (*p* ≥ 0.05).

To evaluate the potency of exosomes in awakening dormant cancer cells, pEOCs, A2780, OVCAR-3, and SKOV-3 cells were placed under serum-free conditions to induce a dormant state [7]. Following this, the cells were incubated with complete conditioned medium containing both exosomes and other non-exosomal constituents (e.g., soluble proteins, vitamins, etc.), as well as with exosomes isolated from this medium. The effects of the non-exosomal fraction were assessed by calculating the difference between the effects of the complete medium and those of the exosomes. The activation of cancer cells was confirmed by increased Ki-67 proliferation antigen expression. The results showed that all cancer cell lines tested regained their proliferative potential more effectively when exposed to exosomes than to the non-exosomal medium constituents. This effect was evident in both young and senescent PMCs and PFBs (Figure 2).

### 3.3. Exosomes Derived from Senescent PMCs and PFBs Are More Effective in Reactivating the Proliferation of Dormant EOC Cells Compared to Exosomes from Younger Cells

**Figure 3:**
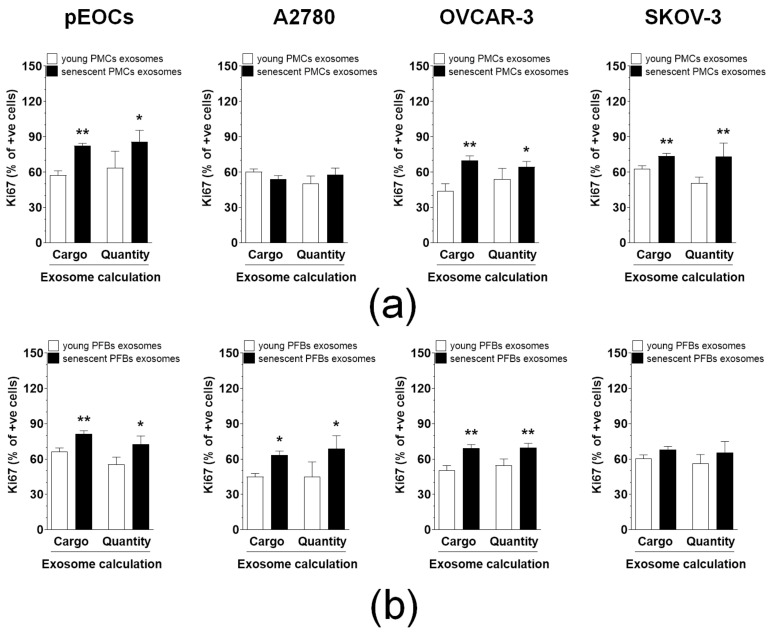
The Influence of exosomes from young and senescent PMCs and PFBs on the awakening of dormant ovarian cancer cells. The expression of Ki-67 was used as a measure of EOC cell activation in response to exosomes derived from PMCs (**a**) and PFBs (**b**). The experiment was conducted in two variants, examining the effects of exosomal composition (cargo) and quantity. Statistical significance was determined with * *p* < 0.05 and ** *p* < 0.01 when comparing exosomes from young cells. Exosomes obtained from PMCs and PFBs of eight different donors were used in the experiments, and the results are presented as mean ± SEM.

The study investigated the ability of exosomes derived from both young and senescent PMCs and PFBs to awaken dormant cancer cells through two experimental variants. In the first variant, referred to as “cargo,” a constant number of exosomes—accounting for the differing production levels of these particles in young and senescent cells—was applied to cancer cells. This approach enables the assessment of the significance of exosome composition rather than their quantity, as the number administered to the recipient cells was standardized. In this setup, exosomes from senescent PMCs increased the percentage of Ki67-positive cells in pEOCs, OVCAR-3, and SKOV-3 cells, but not in A2780 cells (Figure 3a). Conversely, exosomes from senescent PFBs stimulated Ki67 expression in pEOCs, A2780, and OVCAR-3 cells, but not in SKOV-3 cells (Figure 3b).

The second experimental variant, named “quantity,” involved administering exosomes derived from a fixed number of cells—500,000 young and senescent cells—in which the recipient cancer cells received a greater number of exosomes from senescent cells compared to young ones. Under these conditions, exosomes from both senescent PMCs and PFBs reactivated the same dormant cancer cells as observed in the “cargo” variant (Figure 3a,b).

### 3.4. Exosomes from Senescent PMCs and PFBs Show Enhanced Internalization in Recipient Cancer Cells

**Figure 4:**
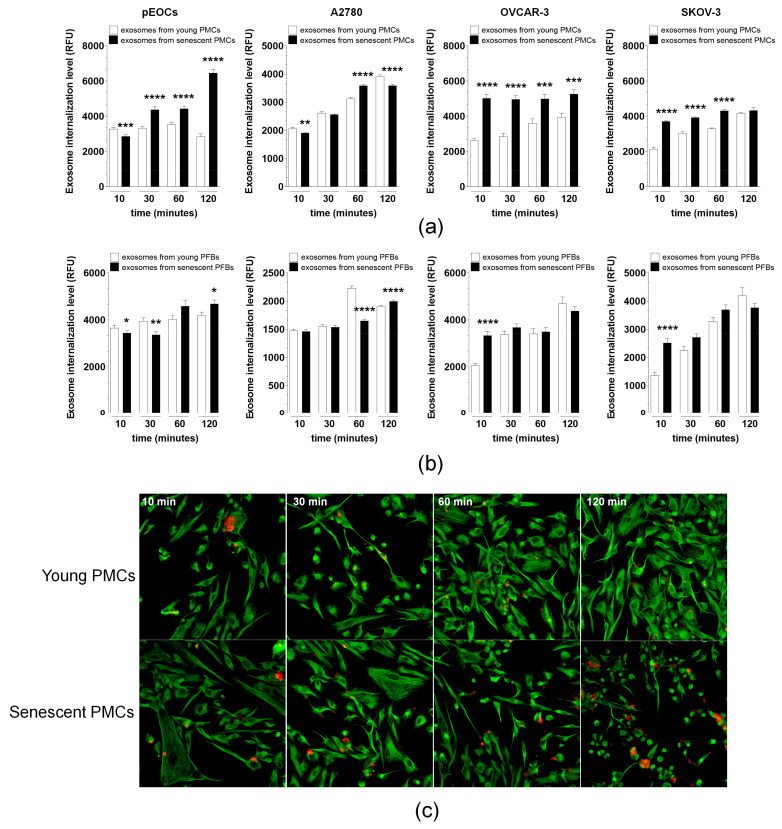
The internalization kinetics of exosomes in recipient cancer cells. (**a**) Time-course analysis of exosome uptake efficiency in pEOCs, A2780, OVCAR-3, and SKOV-3 cells following exposure to PMC-derived exosomes from young (open circles) and senescent (filled circles) cells. Measurements were taken at 10, 30, 60, and 120 min post-administration. (**b**) Corresponding analysis for PFB-derived exosomes. (**c**) Representative fluorescence microscopy images showing the kinetics of CD81-PE-labeled exosome internalization (red) within Cell Tracker Green-stained pEOCs (green) at 10, 30, 60, and 120 min. Merged images demonstrate progressive accumulation of exosomes within the cytoplasm. Magnification ×400; scale bar = 50 μm. * *p* < 0.05, ** *p* < 0.01, *** *p* < 0.001, **** *p* < 0.0001 when comparing exosomes from young vs. senescent cells at each time point. *n* = 8 donors; results reported as mean ± SEM.

A time-course experiment was conducted to compare the efficiency of exosome internalization by recipient cancer cells. The time intervals examined were 10, 30, 60, and 120 min. The results revealed that exosomes from senescent PMCs were internalized by pEOCs at a higher efficiency than those from young cells, particularly 30 min after administration. This trend continued through to the 120 min mark (Figure 4a,c). For OVCAR-3 cells, the internalization of exosomes from senescent PMCs was consistently stronger at every time point measured. In recipient SKOV-3 cells, the internalization of exosomes from senescent cells was more pronounced between 30 and 60 min after addition, while by 120 min, the incorporation of exosomes from both young and senescent cells was comparable. In the case of A2780 cells, exosomes from senescent PMCs were internalized robustly at 60 min, but exosomes from young cells gained an advantage by 120 min of exposure (Figure 4a).

Regarding PFBs, pEOCs internalized exosomes from young cells more efficiently than those from senescent cells at both 10 and 30 min. At 60 min, the incorporation of exosomes from both groups was similar; however, by 120 min after exosome administration, the uptake of particles from senescent cells was greater than that from young cells. In A2780 cells, a similar trend was observed as with PMCs: exosomes from young cells were internalized at a higher efficiency at 60 min, while the uptake of exosomes from senescent cells was stronger at 120 min. Finally, OVCAR-3 and SKOV-3 cells exhibited similar behavior; they internalized exosomes derived from senescent cells at a higher efficacy 10 min post-addition, while the internalization of both types of exosomes became comparable in subsequent measurements (Figure 4b).

### 3.5. Exosomes Derived from Young and Senescent PMCs and PFBs Show Differences in Their Composition

A quantitative comparison was made of three groups of exosome components found in the particles released by both young and senescent PMCs and PFBs. These groups included mRNAs, microRNAs, and proteins. The mRNAs analyzed were IL6, CXCL8, CXCL12, CXCL1, CCL2, TGFB1, TIMP1, SPP1, MMP1, MMP2, and LPA. Notably, there were no differences in the expression levels of these mRNAs between the exosomes derived from young and senescent cells.

**Figure 5:**
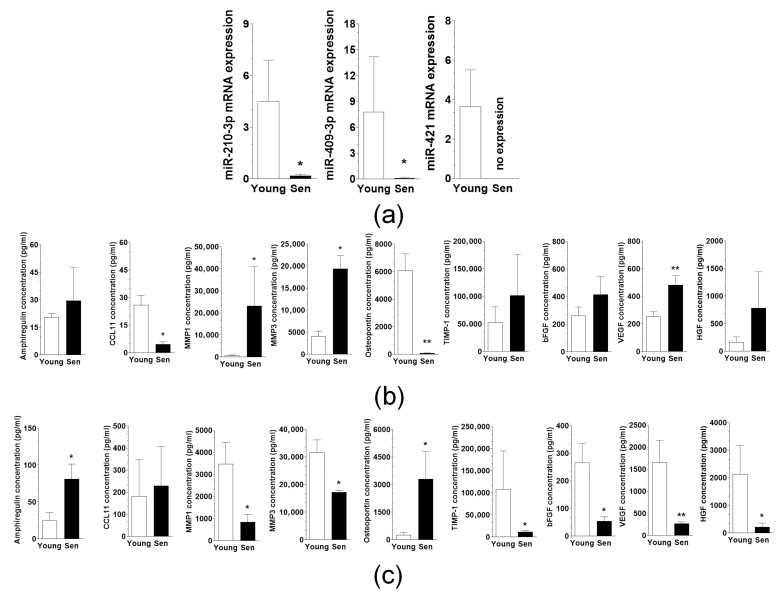
Composition analysis of exosomes. A comparison of microRNA expression changes in exosomes from young and senescent PMCs (**a**). Quantification of exosomal proteins in PMCs (**b**) and PFBs (**c**). * *p* < 0.05, ** *p* < 0.01 indicates significance compared to exosomes from young cells. The experiments utilized exosomes derived from PMCs and PFBs of four different donors, with samples prepared in duplicates. Results are presented as mean ± SEM.

A panel of microRNAs was examined, including hsa-miR-27b-5p, hsa-miR-105-5p, hsa-miR-92a-1-5p, hsa-miR-99a-5p, hsa-miR-409-5p, hsa-miR-4454, hsa-miR-612, hsa-miR-4748, hsa-miR-614, hsa-let-7a-5p, hsa-miR-1299, hsa-miR-3689b-5p, hsa-miR-421, hsa-miR-210-5p, hsa-miR-329-5p, hsa-miR-543, hsa-miR-3689a-5p, hsa-miR-323a-5p, and hsa-miR-3689d. Among these, the expression levels of miR-210-3p, miR-409-3p, and miR-421 were reduced in exosomes derived from senescent PMCs (Figure 5a). In contrast, no microRNAs showed differential expression between exosomes isolated from young and senescent PFBs.

The protein cargo in exosomes was quantified using a customized cytokine array that included the following proteins: amphiregulin, bFGF, CXCL5, CCL11, GRO-1, HGF, IL-6, IL-6R, IL-8, MMP1, MMP3, MMP9, MCP-1, osteopontin, PAI-1, SDF-1, TGF-β1, TIMP-1, u-PA, and VEGF. A comparative analysis of the protein content in these exosomes yielded ambiguous results. In the case of PMCs, senescent cell exosomes displayed an increased concentration of MMP1, MMP3, and VEGF, while showing a decreased concentration of eotaxin-1 and osteopontin (Figure 5b). For PFBs, senescent cell exosomes were characterized by elevated levels of amphiregulin and osteopontin, alongside decreased concentrations of MMP1, MMP3, TIMP1, bFGF, VEGF, and HGF (Figure 5c). Regarding the remaining cytokines, their concentrations varied between cultures; some showed an increase in exosomes from senescent cells, while others exhibited a decrease. Overall, the results were notably patient-specific and applied to both PMCs and PFBs.

### 3.6. Exosomes from Senescent PMCs and PFBs Stimulate the Reinitiation of pEOC Progression

**Figure 6:**
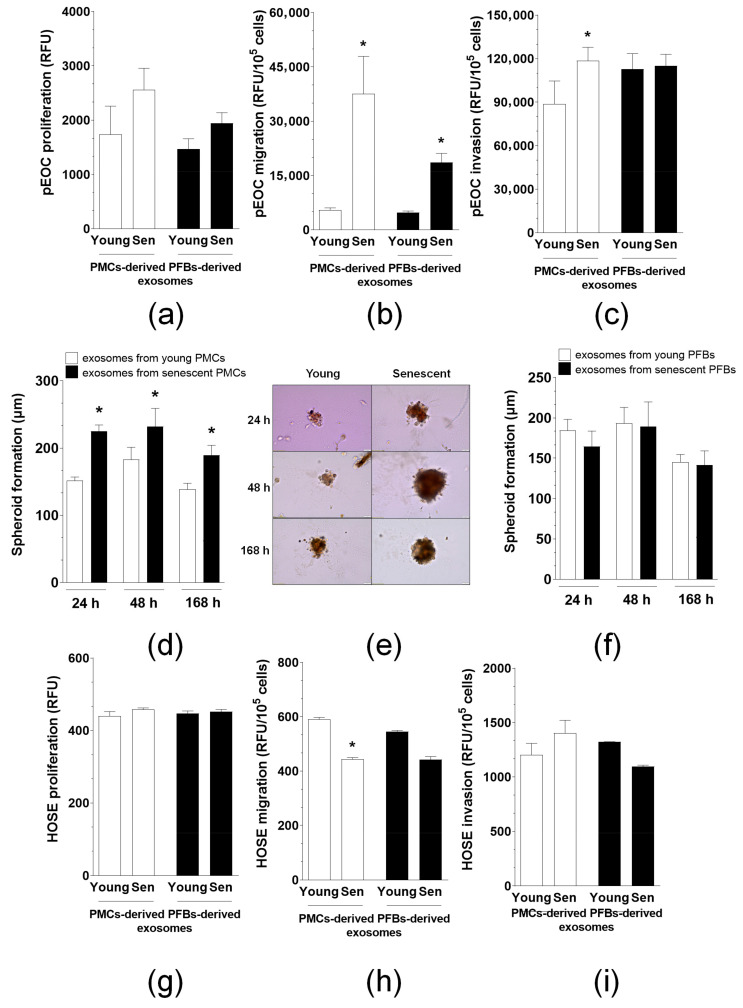
Analysis of awakened cancer cell progression. Proliferation (**a**), migration (**b**), and invasion (**c**) of pEOCs in response to exosomes generated by young and senescent PMCs and PFBs. Kinetics of spheroid formation in pEOCs treated with exosomes from PMCs (**d**) and PFBs (**f**). Representative images of pEOC spheroids following exposure to exosomes from both young and senescent PMCs. Magnification ×200 (**e**). Effects of exosomes on HOSE cell proliferation (**g**), migration (**h**), and invasion (**i**). Significance was determined with * *p* < 0.05 when comparing results to exosomes derived from young cells. The experiments utilized exosomes obtained from PMCs and PFBs of six different donors, and results are presented as mean ± SEM.

Exosomes derived from either senescent PMCs or PFBs did not support the long-term proliferation of pEOCs (Figure 6a). However, we observed that pEOCs yielded the most consistent results in experiments investigating the awakening of dormant cancer cells in response to exosomes derived from PMCs and PFBs. Consequently, further experiments focusing on the reinitiation of cancer cell progression were conducted exclusively with these cells. The studies demonstrated that exosomes generated by senescent PMCs, when applied to pEOCs for 72 h, stimulate the migration, invasion, and spheroid formation of pEOCs (Figure 6b–e). In contrast, exosomes from senescent PFBs promoted migration but failed to support invasion and spheroidogenesis (Figure 6b,c,f).

Interestingly, experiments conducted in HOSE cells, which represent normal ovarian surface epithelium, revealed that their migration in response to exosomes from senescent PMCs was lower than their migration in response to exosomes from young cells. However, when it came to proliferation and invasion, the effects of exosomes from both young and senescent cells were comparable (Figure 6g–i).

### 3.7. Exosomes Derived from Senescent PMCs and PFBs Alter the Transcriptome of Recipient Cancer Cells

**Figure 7:**
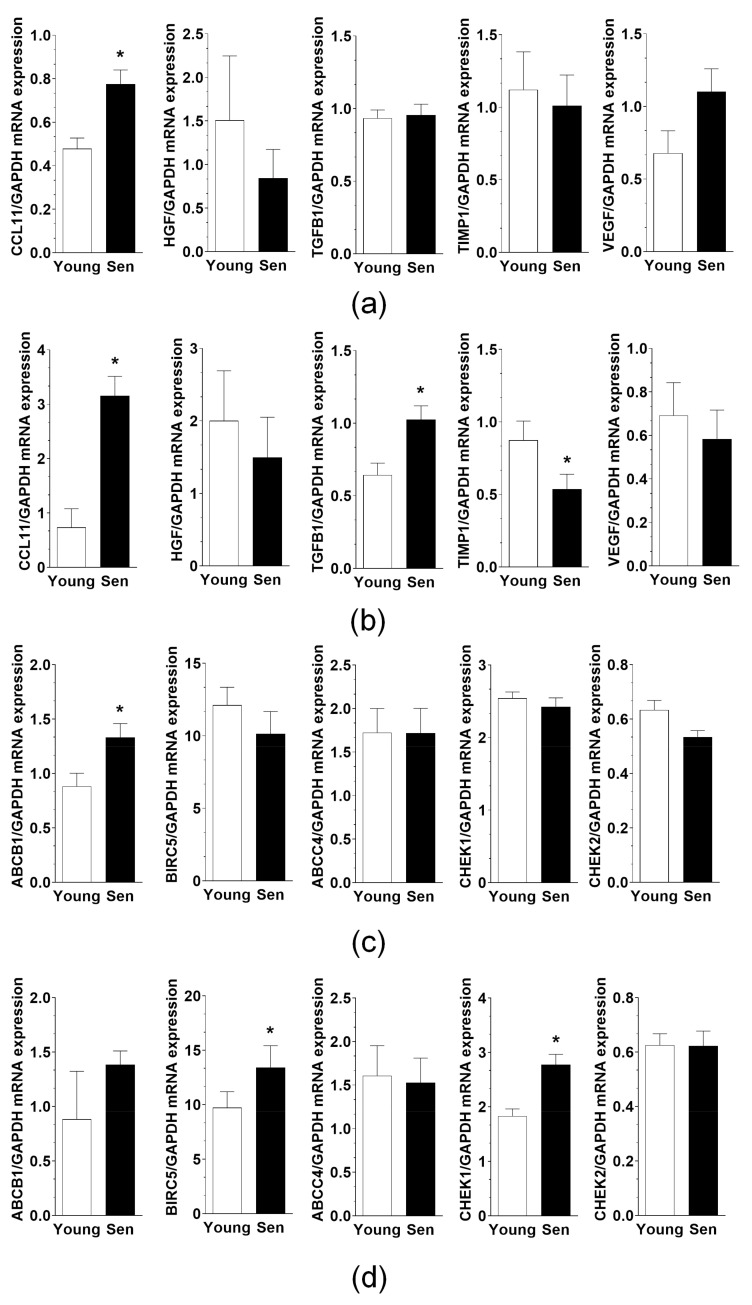
Quantitative analysis of mRNA expression in genes associated with cancer cell progression, and chemoresistance. The expression levels of CCL11, HGF, TGF-β1, TIMP1, and VEGF were analyzed in pEOCs subjected to exosomes from both young (Young) and senescent (Sen) PMCs (**a**) and PFBs (**b**). GAPDH served as the housekeeping gene for normalization. Statistical significance was determined with a threshold of * *p* < 0.05, comparing the results to those derived from exosomes of young cells. Exosomes were collected from PMCs and PFBs from six different donors, and samples for qPCR reactions were prepared in duplicate. The results are presented as mean ± SEM. The expression levels of ABCB1, BIRC5, ABCC4, CHEK1, and CHEK2 were analyzed in pEOCs treated with exosomes derived from both young (Young) and senescent (Sen) PMCs (**c**) and PFBs (**d**). GAPDH served as the housekeeping gene for normalization. Statistical significance was assessed using a threshold of * *p* < 0.05, comparing the results from exosomes of young cells to those from senescent cells. Exosomes were collected from PMCs and PFBs from six different donors, and samples for qPCR reactions were prepared in duplicate. The results are presented as mean ± SEM.

Five arbitrarily selected transcripts related to EOC cell progression were quantified using qRT-PCR to investigate whether exosomes produced by senescent cells and applied to pEOCs for 72 h may influence the transcriptome of recipient cells compared to those secreted by young cells. The transcripts analyzed included CCL11, HGF, TGF-β1, TIMP1, and VEGF. In the case of PMCs, exosomes derived from senescent cells resulted in an upregulation of CCL11 mRNA, while the expression of the other transcripts remained unchanged (Figure 7a). In contrast, exosomes from senescent PFB cells not only increased the expression of CCL11 and TGF-β1 mRNA but also decreased the expression of TIMP1 mRNA (Figure 7b).

In the same experimental settings, the expression of five genes associated with chemoresistance in EOC was quantified. These genes were ABCB1, BIRC5, ABCC4, CHEK1, and CHEK2. Exosomes derived from senescent PMCs led to an up-regulation of ABCB1 mRNA, while the expression levels of the other genes remained unchanged (Figure 7c). In contrast, senescent PFB exosomes resulted in increased expression of BIRC5 and CHEK1 mRNAs, with the other transcripts showing no significant alteration (Figure 7d).

## 4. Discussion

In this study, we investigated our original hypothesis that ovarian cancer recurrence arises from the reactivation of dormant cancer cells, mediated by exosomes released by senescent peritoneal stromal cells. We focused specifically on peritoneal mesothelial cells (PMCs) and peritoneal fibroblasts (PFBs), which compose the peritoneal microenvironment and function as key regulators of peritoneal homeostasis [11,12]. The dormancy model used in this study was based on our previously validated serum starvation protocol [7], which provides a reproducible method for inducing and terminating ovarian cancer cell dormancy in vitro. In that methodological study, we demonstrated that 72 h serum starvation induces reversible G0/G1 arrest accompanied by reduced Ki67 expression and decreased ERK1/2/p38 MAPK activity ratio, without inducing apoptosis or senescence. Serum starvation is a well-established approach for inducing quiescence in ovarian cancer cells, as demonstrated by other independent group [13]. However, we acknowledge that this model represents a simplified approximation of the complex in vivo dormancy state, which involves interactions with the immune system, extracellular matrix, and vascular niche. While Ki67 served as a reliable indicator of proliferative reactivation, it does not fully capture the multifaceted nature of tumor dormancy. Future studies should incorporate additional dormancy-associated markers, such as p27 (a CDK inhibitor that accumulates during G0 arrest and has been validated as a dormancy marker in ovarian cancer), NR2F1 (an orphan nuclear receptor controlling dormancy programs), and DEC2/BHLHE41 (a transcription factor associated with dormancy), to provide a more comprehensive characterization of the dormant state and its reversal. Building on our previous work, in which we demonstrated that carboplatin combined with paclitaxel induces premature senescence in PMCs and PFBs and drives the emergence of a pro-tumorigenic secretory phenotype (SASP) that accelerates EOC progression [6], the present study further investigates whether exosomes generated by these senescent cells contribute to the reactivation of dormant cancer cells. In that earlier work, SASP activity was primarily attributed to soluble proteins. However, recent studies demonstrate that SASP also includes extracellular vesicles, especially exosomes, which may serve as potent paracrine regulators in cancer progression [14,15]. This prompted us to evaluate whether exosomes produced by senescent peritoneal cells contribute to EOC recurrence by reawakening dormant cancer cells [16]. Our findings indicate that senescent PMCs and PFBs produce a markedly higher number of exosomes than young cells, as evidenced by elevated levels of the canonical exosomal markers CD9, CD63, and CD81 [17]. This finding is consistent with previous research conducted on various cellular models [15,18]. The isolated vesicles aligned with the established features of exosomes [19], confirming that our isolation protocol yielded biologically relevant extracellular vesicles. Importantly, exosomes were more effective than non-exosomal constituents of conditioned media (e.g., soluble proteins) in reactivating dormant EOC cells. Notably, a recent report indicates that the pro-tumorigenic exosome secretome from senescent cells differs significantly from the soluble protein components of the SASP [20]. Our findings, showing that exosome-mediated signaling can exhibit greater functional potency than non-exosomal agents, are, however, novel and suggest that exosomes may function as protected carriers of pro-growth cues that remain active in the peritoneal environment, thereby enabling dormant ovarian cancer cells to resume proliferation and contribute to disease recurrence.

To test whether exosome quantity or molecular composition determines their awakening capacity, we employed two experimental variants. In the first experiment, equal numbers of exosomes were applied to dormant cancer cells, enabling evaluation of cargo-specific effects. In the second, exosomes were supplied according to the natural release profiles of senescent and young cells, allowing quantity-based effects to manifest. Our observations clearly indicate that both the number of exosomes and the qualitative nature of their cargo influence dormant cell reactivation. These findings align with previous reports, which indicate that senescent cells not only release increased numbers of exosomes but also undergo selective packaging of bioactive cargo that can modulate the behavior of recipient cells [21,22]. Specifically, the work by Mas-Bargues et al. [21] highlights that both the abundance and molecular composition of vesicles change with cellular senescence, thereby enhancing their functional impact on the microenvironment. Complementarily, Lee et al. [22] describe how regulated cargo sorting during exosome biogenesis determines the potency and specificity of vesicle-mediated signaling. Together, these studies support our observation that dormant EOC cells are influenced by both the quantity and the qualitative nature of exosomes derived from senescent PMCs and PFBs, emphasizing the coordinated adaptation of exosomal production and cargo to promote tumor cell reactivation.

We next examined whether exosomes from senescent cells were internalized more efficiently by recipient EOC cells. In most cell models tested, exosomes from senescent PMCs and PFBs demonstrated enhanced internalization kinetics compared to exosomes from young cells. Increased uptake can amplify downstream biological effects and may represent one mechanism underlying the heightened ability of senescent cell-derived exosomes to awaken dormant cells.

In the next step, we analyzed the molecular composition of exosomes derived from both young and senescent cells. This investigation was informed by prior studies showing that cellular senescence alters the cargo of exosomes, potentially influencing their effects on recipient cells [23,24]. Here observed that protein composition varied among patients and was not uniformly altered in senescent cells. While some pro-invasive and angiogenic proteins, such as MMP1, MMP3, and VEGF, were elevated in exosomes from senescent PMCs, the overall protein expression pattern was inconsistent and did not fully explain the robust biological effects observed. This likely indicates that the protein panel analyzed was too limited to capture the complexity of the senescence-associated exosomal proteome. Future broader proteomic and lipidomic analyses will be necessary to identify dominant mediators of exosome-driven cancer reactivation.

In contrast, microRNA profiling revealed a more consistent pattern. Exosomes derived from senescent PMCs exhibited reduced levels of miR-210-3p, miR-409-3p, and miR-421. These microRNAs have been previously implicated in the repression of genes involved in proliferation, migration, and survival signaling. For example, miR-210-3p regulates hypoxia-responsive pathways, including HIF signaling [25]. miR-409-3p suppresses proliferation, migration, and invasion in certain cancer cell types [26], and miR-421 has been associated with the suppression of cell viability, the delay of the cell cycle, a reduction in glycolysis, and the inhibition of cancer cell migration [27]. The reduced abundance of these microRNAs in exosomes from senescent PMCs may therefore release inhibitory constraints on pathways that promote the reactivation and progression of dormant ovarian cancer cells.

While our compositional analysis revealed reduced levels of miR-210-3p, miR-409-3p, and miR-421 in senescent PMC-derived exosomes, alongside elevated MMP1, MMP3, and VEGF, these findings remain correlative. Establishing direct causality will require functional validation through gain-of-function and loss-of-function experiments. Specifically, future studies should employ miRNA mimics and inhibitors transfected into recipient EOC cells to determine whether restoring these miRNAs abrogates the pro-reactivation effects of senescent exosomes. Similarly, functional blocking with neutralizing antibodies against MMP1, MMP3, and VEGF, or with small-molecule inhibitors, would help determine whether these proteins are necessary mediators of exosome-induced dormancy escape. Such mechanistic studies represent an important direction for future investigation.

Functionally, exosomes from senescent PMCs and PFBs stimulated migration, invasion, and spheroid formation of primary EOC cells, indicating that once awakened, these cells not only resume proliferation but also regain metastatic and survival capabilities. The observed upregulation of CCL11 (a mediator of proliferation and migration/invasion of EOC cells [28]), TGF-β1 (a mediator of epithelial–mesenchymal transition [29]), and chemoresistance-associated transcripts such as ABCB1 [30], BIRC5 [31], and CHEK1 [32] further supports the notion that senescent cell-derived exosomes help initiate transcriptional programs that favor recurrence and treatment resistance. This is particularly relevant given that recurrent EOC is characteristically chemoresistant and clinically difficult to control [33]. Notably, normal HOSE cells did not display increased activation or aggressive behavior in response to senescent cell-derived exosomes. These data indicate that the pro-cancer effects of exosomal signaling are selective for malignant cells and do not extend to normal ovarian epithelial cells, which may have implications for therapeutic targeting.

All findings in the present study are based on in vitro experiments, which limits their direct translational relevance. The peritoneal microenvironment in vivo involves complex interactions between multiple cell types, immune cells, extracellular matrix components, and vascular networks that cannot be fully recapitulated in cell culture. However, to enhance the physiological relevance of our observations, we employed a 3D spheroid model cultured in Matrigel, which more closely recapitulates in vivo conditions compared to standard 2D cultures and is widely recognized as an intermediate model bridging classical in vitro studies and animal experiments. Furthermore, the use of primary EOC cells (pEOCs) derived from chemotherapy-naïve patients with high-grade serous tumors strengthens the translational significance of our findings. Future studies should therefore validate these findings using in vivo models, such as xenograft models with dormant EOC cells and the administration of senescent cell-derived exosomes, or murine models with chemotherapy-induced peritoneal cell senescence and monitoring of tumor recurrence. Analysis of exosomes from peritoneal fluid of ovarian cancer patients before and after chemotherapy would also provide valuable clinical correlation.

## 5. Conclusions

Taken together, our findings indicate that senescent PMCs and PFBs contribute to the formation of a relapse-permissive peritoneal microenvironment by producing exosomes capable of reactivating dormant cancer cells and promoting their subsequent malignant progression. These observations support a model in which chemotherapy-induced stromal senescence paradoxically fosters future disease recurrence. Although these findings are based on in vitro experiments, the use of primary patient-derived EOC cells and a 3D spheroid model cultured in Matrigel enhances their physiological relevance. In vivo validation represents a planned direction for future research. It will be essential to confirm the translational potential of these observations for understanding how therapy-induced changes in the peritoneal microenvironment contribute to ovarian cancer relapse.

## Data Availability

The data underlying this article will be shared on reasonable request to the corresponding author.

## Abbreviations

The following abbreviations are used in this manuscript:

EOC	Epithelial ovarian cancer
HOSEpiC	Human ovarian surface epithelial cells
PFBs	Peritoneal fibroblasts
PMCs	Peritoneal mesothelial cells
SASP	The Senescence-Associated Secretory Phenotype
